# Radiation Chronotherapy in Prostate Cancer: Does Time of Day of Radiation Treatment Influence Disease Outcome or Symptom Burden? †

**DOI:** 10.3390/cancers17152441

**Published:** 2025-07-23

**Authors:** Greeshma Rajeev-Kumar, Aoi Shimomura, Yan Che, Christopher Stepaniak, Stanley L. Liauw

**Affiliations:** 1 Department of Radiation and Cellular Oncology, University of Chicago, Chicago, IL 60637, USA; greeshma.rajeev-kumar@uchicagomedicine.org (G.R.-K.); aoi.shimomura@cshs.org (A.S.); christopher.stepaniak@uchicagomedicine.org (C.S.); 2 Biostatistics Laboratory, University of Chicago, 5758 S Maryland Ave, Chicago, IL 60637, USA; yche@bsd.uchicago.edu

**Keywords:** prostate cancer, radiation therapy, circadian rhythm, chronoradiotherapy

## Abstract

Background: Circadian rhythms regulate critical cellular processes, including DNA repair and cell cycle dynamics, potentially influencing the effectiveness of radiotherapy (RT). This study evaluated whether RT timing impacts clinical outcomes and symptom burden in prostate cancer patients. Patients/Methods: This retrospective study (*n* = 336, median follow-up 55 months) included men who received curative intent external beam RT between 2010 and 2019 (median age 69, 69% black, median PSA 11.3, 40% with Gleason 8–10). Treatment times (TTs) were averaged and analyzed by quartile/median. Outcomes included freedom from biochemical failure (FFBF) and distant metastasis (FFDM), GI and GU toxicity, and quality of life (QOL). Subgroup analyses by race and hormone therapy status were performed. Results: Across the overall cohort, TT was not associated with FFBF or FFDM. However, in white men, earlier TTs were significantly associated with higher 5-year FFBF (89% vs. 67%, *p* = 0.0139) and FFDM (93% vs. 72%, *p* = 0.0268). In the multivariate analysis (MVA), TT was not associated with FFBF or FFDM for all men, but in white men, earlier TT was associated with improved FFBF (HR 2.8, *p* = 0.06) in a model also including risk category (*p* = 0.21). Overall, no significant differences were observed for grade 2–3+ toxicity and TT. Trends for inferior QOL, and worse grade 2+ (*p* = 0.2) and 3+ GU toxicity (*p* = 0.1) were observed for later TTs. In white men, bowel, urinary continence, and irritative/obstructive urinary QOL were worse with later TTs (*p* < 0.05). Conclusions: TT may influence clinical outcomes and symptom burden, particularly in white men. These findings underscore the potential of chronoradiotherapy as a personalized treatment strategy and highlight the need for prospective trials.

## Simple Summary

Radiation treatment time may impact radiation response differently by race. In this retrospective single-institutional study of 336 men with prostate cancer treated with EBRT, white men treated earlier in the day had better freedom from biochemical failure and distant metastasis. No clear differences in toxicity or quality of life were appreciated overall, but worse quality of life was observed for later treatment times in white men.

## 1. Introduction

Circadian rhythms regulate various physiological processes, including cellular mechanisms related to radiation response, and can vary over the day [1,2]. Cancer cells differ from normal cells in that they may exhibit disrupted circadian rhythms [3]. Aligning cancer treatments to patients’ circadian rhythms can be beneficial in maximizing efficacy and minimizing toxicities. Chronoradiotherapy therefore has emerged as a potential treatment strategy, given that radiation therapy (RT) delivered at certain times may have a differential effect on tumor versus normal cells, and allow for optimization of the therapeutic ratio.

Studies suggest that delivering RT at times when DNA repair in normal cells is most active may reduce toxicity, whereas cancer may be more vulnerable at other times due to altered or less effective repair pathways. Individual variations in circadian genes (such as PER, BMAL1, and CLOCK) might influence the response to treatment [2,4]. Circadian rhythm has been shown to differ by race. Studies indicate that specific genetic polymorphisms, particularly within the *CLOCK* gene, are more prevalent in certain populations. For example, African Americans are reported to have higher frequencies of certain polymorphisms linked to delayed circadian phase (delayed sleep onset), potentially contributing to circadian misalignment [5].

Preclinical models have helped us understand the biological underpinnings and genes involved in regulating the circadian rhythm. A carcinogenic effect of clock disruption has also been established in mouse models [2,4]. However, clinical studies in cancer are more limited, particularly within prostate cancer. A retrospective study from Taiwan of 409 prostate cancer patients evaluated whether RT delivery before 5 PM, versus after 5 PM, was associated with acute toxicity or tumor control. Evening RT was found to be associated with worse GI toxicities and biochemical failure-free survival in men with T2b-T3 disease [1]. Another retrospective study from Asia evaluated the effect of proton beam therapy (PBT) on lower urinary tract symptoms (LUTSs) when administered at different times of the day. Morning PBT was associated with an improvement in LUTS and quality of life as compared to treatment around noon or late afternoon [6]. However, studies on other disease sites, such as breast, cervical, and lung cancer, suggest that morning treatments may be associated with worse toxicities [7,8,9].

We explored whether time of day was associated with disease outcome and recovery in a cohort of men at a single institution treated with RT for prostate cancer. We performed a subset analysis by race given the differences in circadian rhythm fluctuations between black and white people.

## 2. Methods

We identified 336 men treated with curative intent external beam RT between 2010 and 2019 [10]. Demographic and treatment information were available in prospectively defined fields within an IRB-approved database. Table 1 summarizes the cohort with descriptive statistics. Of the patients, 227 men (69%) were black, 90 (27%) were white, and 19 were other (5%). The median age was 69, and median PSA was 11.3. Median RT dose was 78 Gy in 39 fractions. Pelvic lymph nodes were included in the treatment volume in 40% of men, and 244 men (73%) received concurrent androgen deprivation therapy (ADT) based on disease risk classification at clinician discretion, for a median duration of 17 months, typically after a 2-month neoadjuvant course. Twenty men (6%) were treated with a brachytherapy boost (median dose, 110 Gy) following external beam RT to the pelvic lymph nodes and prostate (45 Gy).

The time of radiation treatments was largely driven by patient preference. Patients generally had treatment at the same time of day for their entire course of treatment. Treatment time data was extracted from the radiation management software (Aria Oncology Information System, version 15, Varian, Palo Alto, CA, USA). Average treatment time was calculated according to the average time of each patient’s daily treatments between 6:00 a.m. and 5:30 p.m. In order to calculate average treatment time, data on the delivery times for each beam for a particular patient were obtained, and the earliest beam-on time was recorded. Start time was converted into seconds past midnight, averaged across all treatments, and then converted back to a clock time. For analysis, average time of treatment was separated into quartiles and by median, based on distribution.

Patients completed the Expanded Prostate Cancer Index Composite (EPIC-26) quality of life survey (QOL) at initial consultation and each follow-up after completion of RT. Disease outcomes including freedom from biochemical failure (FFBF) and distant metastasis (FFDM) were estimated using the Kaplan–Meier method, and covariates were tested using the log-rank test. Biochemical failure was defined according to the Phoenix criteria as the elevation of prostate-specific antigen (PSA) to nadir plus 2.0 ng/mL. RTOG and CTC gastrointestinal (GI) and genitourinary (GU) toxicity, and patient-reported QOL were analyzed on univariate and multivariable analyses (MVA). The MVA included NCCN risk category and average treatment time. Statistical significance was established at a *p*-value less than 0.05. Subdistribution hazard ratios (sHRs) were estimated using the Fine–Gray competing risks model to assess toxicities. Proportional hazard assumptions were evaluated using model diagnostics. Subgroup analyses by race were performed. All analyses were conducted using JMP (version 14) and R (version 4.2.1).

## 3. Results

The median average treatment time was 10:47 a.m. Treatment time by quartile was 6:37 a.m.–9:11 a.m. (1), 9:12 a.m.–10:46 a.m. (2), 10:47 a.m.–12:41 p.m. (3), and 12:42 p.m.–5:12 p.m. (4). There were no imbalances in NCCN risk category or race across treatment times.

### 3.1. Disease Outcome

**Figure 1:**
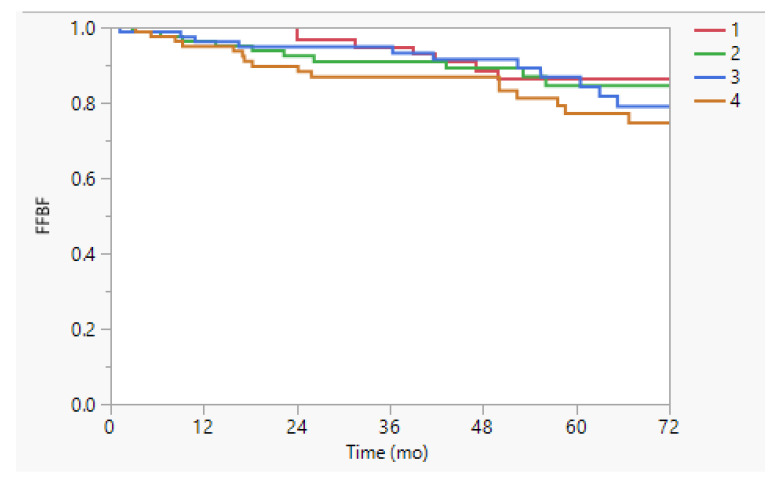
Freedom from biochemical failure (FFBF) stratified by time of treatment quartile. The 5-y FFBF was 86%, 85%, 87%, and 77% (*p* = 0.61) in the overall cohort for treatment quartiles 1 (earliest time of day) to 4 (latest time of day).

At a median follow up of 55 months, the 5-year FFBF was 84% and 5-year FFDM was 91%. Table 2 presents the results of the UVA for the cumulative incidence of biochemical failure and distant metastasis. Outcomes were not different according to treatment time when stratified by either quartile or by median (all *p* > 0.1), in the overall cohort. There were no differences observed in FFBF (*p* = 0.61, Figure 1) or FFDM by treatment quartile (*p* = 0.51).

**Figure A1:**
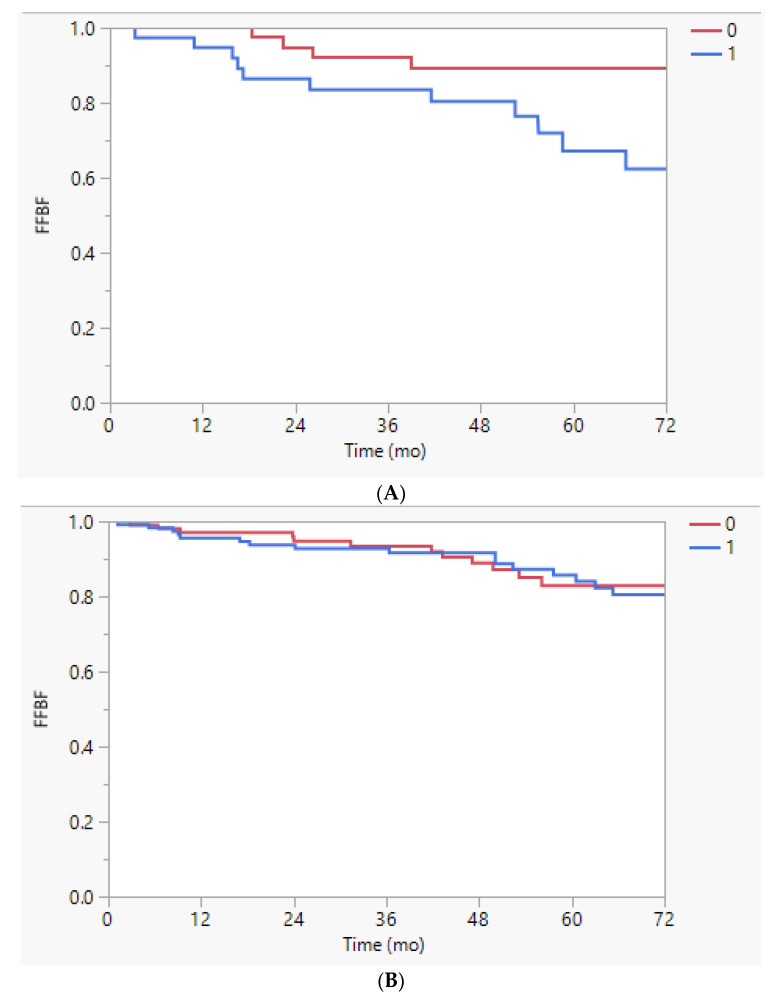
(**A**). Freedom from biochemical failure (FFBF) stratified by time of treatment earlier (0) or later (1) than the median time of 10:47 a.m. in white men, *p* = 0.0139. (**B**). Freedom from biochemical failure (FFBF) stratified by time of treatment earlier (0) or later (1) than the median time of 10:47 a.m. in black men, *p* = 0.50.

**Figure A2:**
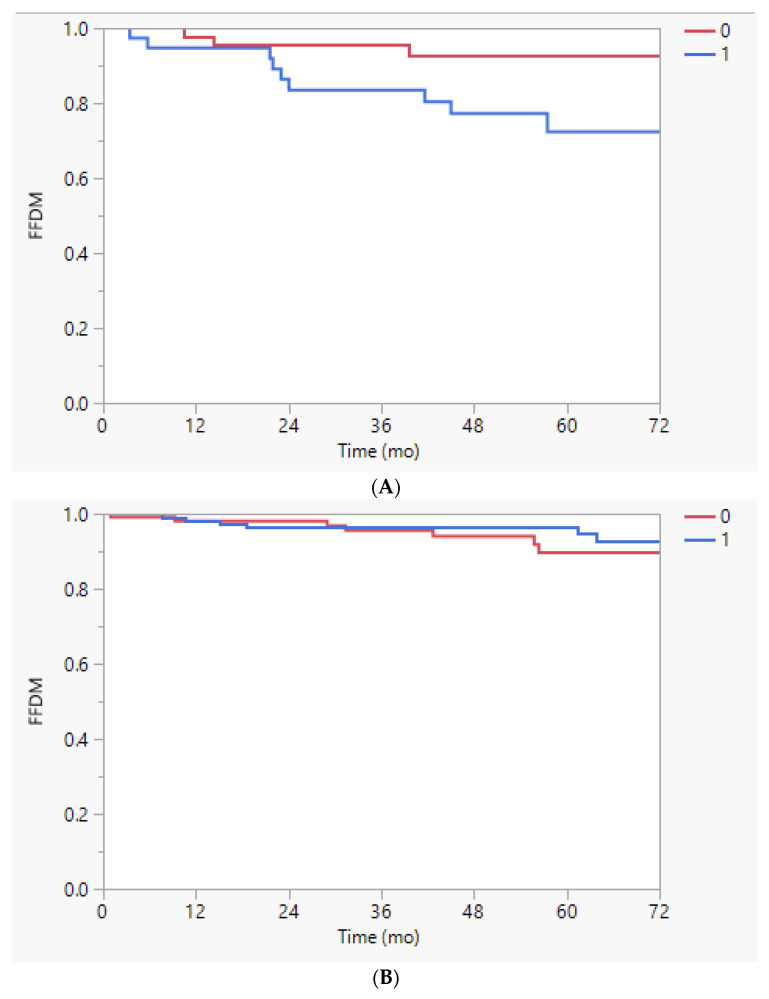
(**A**). Freedom from distant metastasis (FFDM) stratified by time of treatment earlier (0) or later (1) than the median time of 10:47 a.m. in white men, *p* = 0.0268. (**B**). Freedom from distant metastasis (FFDM) stratified by time of treatment earlier (0) or later (1) than the median time of 10:47 a.m. in black men, *p* = 0.85.

However, on the subset analysis by race, white men treated in the first half of the day had better FFBF (89% vs. 67%, *p* = 0.0139) and FFDM (93% vs. 72%, *p* = 0.0268) than those treated in the second half; this difference was not observed for black men (5-y FFBF 83% vs. 86%, *p* = 0.50; 5-y FFDM 90% vs. 96%, *p* = 0.85; Appendix A Figure A1 and Figure A2).

The crude rate of BF in white men was 18%; by quartile of average treatment time, it was 5%, 10%, 28%, and 32%, respectively (*p* = 0.04), whereas in black men, it was, 14%, 17%, 18%, and 21% (*p* = 0.81, Table 3). Differences in BF across treatment time were not observed in a subset analysis by age (≥69, *p* = 0.35; <69, *p* = 0.24) or by NCCN risk category (low, *p* = 0.53; favorable intermediate, *p* = 0.28; unfavorable intermediate, *p* = 0.39; high, *p* = 0.21). However, differences in BF were observed in a subset analysis by ADT, in which men on ADT appeared to be preferentially impacted by treatment time. BF occurred in 17% of men on ADT (*n* = 244); by quartile of average treatment time, the rate of failure was 9%, 14%, 19%, and 29% (*p* = 0.0326). By comparison, BF occurred in 18% of men not on ADT (*n* = 89); by quartile of treatment time, the rate of failure was 19%, 17%, 21%, and 16% (*p* = 0.97). Differences by time of treatment were observed for white men on ADT (5-y FFBF 89% vs. 65%, *p* = 0.0211) rather than for black men on ADT (5-y FFBF 81% vs. 86%, *p* = 0.96).

### 3.2. Quality of Life and Toxicity

A univariate analysis (Table 4) was performed for each domain of patient-reported global QOL (urinary continence, urinary irritation/obstruction, bowel function, and sexual function). In the overall cohort, treatment time was not associated with QOL, although there were trends observed for later treatment time and urinary incontinence (*p* = 0.16 for quartile 4), and urinary irritative/obstruction (*p* = 0.09 for quartile 3). There was no difference observed in any QOL domain and race on the UVA. Other covariates tested which were associated with QOL included diabetes (urinary irritative/obstructive, *p* = 0.02; sexual, *p* = 0.01; NCCN risk group, *p* < 0.001), ADT use (sexual, *p* = 0.002), and age (sexual, *p* = 0.02).

On the subset analysis by race, associations in white men for treatment time were stronger. Later treatment time was associated with worse urinary incontinence (−14.9, *p* = 0.009 for quartile 3; −5.6, *p* = 0.07 for quartile 4), urinary irritation/obstruction (−6.49, *p* = 0.08 for quartile 4), bowel function (−8.4, *p* = 0.004 for quartile 3; −5.6, *p* = 0.05 for quartile 4), and sexual function (−15.7, *p* = 0.06 for quartile 3; −12.9, *p* = 0.10 for quartile 4). These differences were not observed in the subset of black men (all *p* > 0.2) with the exception of the sexual domain (higher health with later treatment, +8.49, *p* = 0.04 for quartile 4).

No significant differences were observed for late grade 2–3+ GI toxicity and treatment time or race. A trend for worse grade 2+ GU toxicity (*p* = 0.199 for quartile 4) was observed in the entire cohort, but there was no difference by race and GU toxicity. There were too few grade 3+ GU toxicity events to conduct meaningful analyses.

On MVA (Table 5), treatment time was not associated with FFBF or FFDM when included in a model with NCCN risk category, although there was a statistical trend (*p* < 0.2) for treatment time. Risk category was chosen for inclusion in the model rather than other disease risk factors associated with outcome on UVA (PSA, Gleason score, T stage) in order to avoid overfitting the model with a limited number of events. Hazard ratios (HRs) could not be estimated for several comparisons due to zero events in one or more groups. On an MVA including white men only, treatment time was associated with FFBF (HR 2.8, *p* = 0.0583) in a model including risk category (*p* = 0.21); a trend was also observed for treatment time with FFDM (*p* = 0.09) when tested alongside risk category (*p* = 0.10). In black men, there was no association with treatment time and FFBF or FFDM.

## 4. Discussion

In this exploratory analysis, we observed that the timing of RT may influence disease outcomes and symptom burden in men with prostate cancer, with notable racial differences. Specifically, white men treated earlier in the day experienced fewer biochemical failures and distant metastases, while later treatment time was associated with worse urinary, bowel, and sexual QOL.

Earlier reports described the potential impact of circadian rhythm on cancer treatment outcome [11,12]. Circadian rhythms govern a vast array of cellular processes, including DNA repair, apoptosis, cell cycle progression, oxidative stress response, and immune modulation, all of which are directly relevant to radiation sensitivity [2,4,11,12]. The transcription of DNA repair genes including ATM, RAD51, and BRCA1 fluctuates with the time of day, influenced by clock-regulated transcription factors such as the CLOCK and PER genes. Studies in mice have shown that radiation delivered when these repair pathways are most active in normal tissues reduces toxicity, while tumor cells—often harboring disrupted circadian oscillations—remain more vulnerable. Clinical studies have suggested improved outcomes with morning radiation therapy for skin cancer [13], lung cancer [14], and cervical cancer [15], consistent with our finding of improved disease outcomes in white men treated with RT for prostate cancer. Several studies have also investigated the impact of treatment time on toxicities. In head and neck cancer, patients randomized to receive morning radiation had lower rates of mucositis than patients receiving afternoon radiation [16]. In contrast, a randomized study of cervical cancer patients showed higher rates of RT-induced diarrhea in the morning cohort [8]. Breast cancer studies have demonstrated contradicting findings, with higher rates of acute toxicities with afternoon treatments and late toxicities with morning treatments [7,17]. In prostate cancer, a retrospective study of 409 men from Taiwan demonstrated less acute GI toxicities and improved biochemical failure-free survival among patients with more advanced disease treated before 5PM. An interaction between age and treatment time was also reported; older patients over the age of 70 had worse late proctitis if treated in the evening. Finally, a recent meta-analysis evaluating immunotherapy for various types of cancer involving 1663 patients showed that earlier treatment had roughly twice the response rate and half the risk of death [18], while a recently presented randomized trial of immunochemotherapy in 210 patients with metastatic non-small cell lung cancer confirmed that earlier treatment significantly improved response rate, progression-free survival, and overall survival [19]. The circadian influence on the immune system intersects mechanistically with RT. CD8+ T-cell activity, cytokine release, and antigen present all-display circadian rhythmicity, peaking in the morning. Radiation enhances these immune responses via STING activation and increased tumor antigen visibility, suggesting that morning RT may leverage both enhanced DNA repair in normal tissue and heightened immune activity.

Whether sensitivity to treatment time of day could vary by race requires further study, but both outcomes after RT for prostate cancer and circadian regulation have been shown to vary by race [20]. Comparative studies suggest black men have inherently more favorable responses to radiation and hormonal therapies. In a meta-analysis of seven RTOG/NRG randomized trials of definitive RT, black men had lower rates of biochemical recurrence, distant metastasis, and cancer mortality than white men [21]. In the equal-access healthcare setting of the Veteran Affairs Health System, black men similarly had a lower risk of distant metastasis and cancer-specific mortality [22]. This has been described to be potentially related to underlying genetic differences, including sensitivity to hormonal therapy [23] or DNA damage response by race [24]. Comparisons regarding toxicity and quality of life after RT are also suggestive of differences by race [25] although this has not been widely supported in single-institution studies with prostate cancer [26,27,28]. In a multicenter, prospective study utilizing the Expanded Prostate Cancer Index Composite (EPIC-50), black men reported less bowel bother after RT than white men [29]. Finally, in a study of cancers following radiation therapy in the Veterans Affairs Health System, black men were significantly less likely (HR 0.76) to have a secondary cancer than white men [30]. Apart from the potential contribution of radiation sensitivity, black men may also have differences in circadian regulation. African Americans are known to have a higher prevalence of CLOCK gene polymorphisms, including variants associated with delayed circadian phase and altered circadian amplitude [5,31]. More recently, alterations in other specific circadian rhythm gene pathways, specifically PER1 and PER3, were found to be associated with an increased risk of prostate cancer among all men [32,33]. Race-based polymorphisms may lead to greater circadian misalignment [34], which in turn, could blunt the benefit of timing-based RT strategies.

Based on an exploratory univariate subset analysis, it is possible that the observed differences in outcome by treatment time were enhanced or facilitated by the administration of hormonal therapy. ADT has been shown to alter radiosensitivity, partly by influencing cell cycle dynamics and DNA repair pathways. Given that androgen signaling also intersects with circadian genes, including PER1 and CLOCK, it is conceivable that hormonal therapy could amplify the effects of circadian variation in RT delivery.

A few important limitations deserve attention. As a retrospective analysis, the associations that have been found cannot infer causality. Imbalances in the comparison arms, including only 27% white men in whom differences by treatment time were most prominently demonstrated, could influence the study results, including the chance of reporting a spurious finding. Despite not identifying any obvious differences in patient or disease characteristics by treatment time of day, meaningful differences may have existed according to treatment time that may have favored morning treatments (e.g., patient factors such as higher socioeconomic status, greater likelihood of retirement from employment, and less medical comorbidity, or treatment-related operational factors). We performed a multivariate analysis adjusting for treatment characteristics to attempt to address imbalances, but residual confounding is possible and the model output is further limited by the relatively small number of events. We did not observe associations in the overall cohort and only identified an association with treatment time and disease outcome or QOL in patient subsets. However, our univariate findings with biochemical failure were supported by the multivariate analysis, and the finding that treatment time was associated with both disease outcome as well as QOL in the same subset of white men would seem to support the existence of a common biologic rationale. Our findings, derived from a reasonably large cohort of men with precise information on treatment time and a detailed prospective collection of patient-reported QOL, are unique and potentially clinically impactful. Ultimately, further studies are necessary to verify our hypothesis-generating findings, including in racially diverse populations, before implementing any change in clinical practice.

Although studies support the concept of chronoradiotherapy, the translation into clinical practice is still in its infancy. As described, most clinical data come from small-scale studies with limited power. Circadian rhythms can vary significantly between individuals, influenced by factors like age, lifestyle, and genetic predisposition. Randomized controlled trials are ideal to determine the optimal timing for prostate cancer radiation therapy. Gene expression data would also be valuable to better understand circadian rhythm biology and the mechanisms impacting outcomes by treatment time. Advances in biomarkers of circadian rhythms may help personalize treatment schedules based on an individual’s biological clock. Incorporating genetic profiling and wearable technologies to monitor circadian cycles in real time could refine the timing of RT delivery for better outcomes.

## 5. Conclusions

In this cohort of 336 men treated with curative-intent radiation therapy for prostate cancer, radiation treatment time of day was not significantly associated with disease outcome, toxicity, or patient-reported QOL in the overall group. However, later treatment times were associated with worse disease outcomes and patient-reported QOL in white men. Further study is warranted to study the impact of treatment time on radiotherapy outcomes, including whether differences may exist by race.

## Figures and Tables

**Table 1:** Baseline patient and treatment characteristics.

	Overall Cohort Median (IQR) or N (%)	Median (IQR) or N (%) for Treatment Quartiles 1–2	Median (IQR) or N (%) for Treatment Quartiles 3–4	*p*-Value
Age	69 (64–74)	70 (63–74)	69 (64–74)	1.0
Race (*n* = 334) Asian Black Hispanic White Other	6 (2%) 230 (69%) 7 (2%) 90 (27%) 1 (0%)	3 (2%) 110 (65%) 4 (2%) 50 (30%) 1 (1%)	3 (2%) 120 (72%) 3 (2%) 40 (24%) 0 (0%)	0.54
Gleason Score (*n* = 335) 6 7 8 9 10	24 (7%) 177 (53%) 64 (19%) 65 (19%) 4 (1%)	14 (8%) 87 (52%) 33 (20%) 32 (19%) 2 (1%)	11 (7%) 90 (54%) 31 (19%) 33 (20%) 2 (1%)	0.89
Clinical T-stage (*n* = 324) T1-T2a T2b-2c T3-4 Tx	204 (63%) 37 (11%) 78 (24%) 2 (1%)	110 (68%) 17 (11%) 33 (20%) 2 (1%)	94 (58%) 20 (12%) 45 (28%) 0 (0%)	0.23
PSA	11.3 (6.6–21.9)	10.8 (7–20)	12.3 (7–25)	0.13
Risk Group (*n* = 326) Low Favorable Intermediate Unfavorable Intermediate High	11 (3%) 29 (9%) 97 (30%) 189 (58%)	5 (3%) 17 (10%) 48 (29%) 92 (57%)	6 (4%) 12 (7%) 49 (30%) 97 (59%)	0.78
Hormone Therapy (*n* = 333) Yes No	244 (73%) 89 (27%)	129 (77%) 39 (23%)	115 (70%) 50 (30%)	0.14
Radiation Dose	78 Gy/39 fx	78 Gy/39 fx	78 Gy/39 fx	0.26
Brachytherapy boost	20 (6%)	10 (6%)	10 (6%)	0.98
Median Follow-up	55 months	53 months	59 months	0.0054

IQR = interquartile range

**Table 2:** Univariate analysis for cumulative incidence of biochemical failure and distant metastasis, by Fine–Gray analysis.

	5-Year FFBF	*p*-Value	5-Year FFDM	*p*-Value
Race Black vs. white/other	84% vs. 81%	0.75	93% vs. 85%	0.08
Age 69+ years vs. <69 years	83% vs. 84%	0.59	90% vs. 92%	0.74
NCCN risk group Low/fav int/unfav int/high	91/86/91/78%	0.16	100/100/99/84%	0.0008
PSA 11+ vs. <11	76% vs. 91%	0.0004	90% vs. 92%	0.74
T stage T2b+ vs. <T2b	74% vs. 88%	0.0007	81% vs. 95%	<0.0001
Gleason score Gleason 6–7 vs. 8–10	89% vs. 74%	0.0139	97% vs. 81%	<0.0001
Treatment time 1st/2nd/3rd/4th quartile Before vs. after median	86/85/87/77% 85% vs. 82%	0.61 0.32	90/92/91/89% 91% vs. 91%	0.51 0.14
Treatment time and race Black men, before vs. after median White men, before vs. after median	83% vs. 86% 89% vs. 67%	0.50 0.0139	90% vs. 96% 93% vs. 72%	0.85 0.0268
Brachytherapy boost Yes vs. no	100% vs. 83%	0.07	100% vs. 90%	0.19
Hormonal therapy Yes vs. no	82% vs. 87%	0.68	88% vs. 97%	0.0035

**Table 3:** Crude rate of biochemical failure by race and quartile of average treatment time.

	Black Men (*n* = 230)	White Men (*n* = 90)
Quartile 1	8/58 (14%)	1/21 (5%)
Quartile 2	9/52 (17%)	3/29 (10%)
Quartile 3	11/62 (18%)	5/18 (28%)
Quartile 4	12/58 (21%)	7/22 (32%)
*p*-value	0.81	0.04

**Table 4:** Univariate Analysis for quality-of-life endpoints for the overall cohort and by race. Beta refers to the change in outcomes associated with a one-unit change in the predictor.

		Overall		White Men		Black Men	
	Variable	Beta (SE)	*p*-Value	Beta (SE)	*p*-Value	Beta (SE)	*p*-Value
Urinary incontinence	Intercept	86.8 (1.93)	<0.001	93.1 (2.13)	<0.001	84.3 (2.46)	<0.001
	Quartile 2	−2.19 (2.62)	0.40	−5.67 (3.76)	0.13	−0.96 (3.37)	0.78
	Quartile 3	−1.33 (2.79)	0.63	−14.9 (5.68)	0.009	3.47 (3.21)	0.28
	Quartile 4	−3.74	0.16	−5.60 (3.12)	0.07	−2.46 (3.35)	0.46
Urinary obstruction	Intercept	82.3 (1.37)	<0.001	83.8 (2.78)	<0.001	81.7 (1.64)	<0.001
	Quartile 2	−1.05 (1.87)	0.57	−0.49 (3.40)	0.89	−1.94 (2.39)	0.42
	Quartile 3	−3.54 (2.07)	0.09	−3.47 (3.71)	0.35	−3.69 (2.57)	0.15
	Quartile 4	−2.18 (1.93)	0.26	−6.49 (3.75)	0.08	−0.28 (2.27)	0.90
Bowel function	Intercept	89.7 (0.95)	<0.001	90.9 (1.46)	<0.001	89.5 (1.20)	<0.001
	Quartile 2	−1.32 (1.45)	0.36	−1.69 (2.24)	0.45	−1.85 (1.98)	0.35
	Quartile 3	−0.72 (1.38)	0.60	−8.41 (2.90)	0.004	1.56 (1.56)	0.32
	Quartile 4	−1.69 (1.45)	0.24	−5.64 (2.89)	0.05	−0.13 (1.68)	0.94
Sexual function	Intercept	23.9 (2.78)	<0.001	27.8 (7.11)	<0.001	23.2 (2.95)	<0.001
	Quartile 2	−3.98 (3.72)	0.28	−12.4 (8.25)	0.13	−1.87 (4.41)	0.67
	Quartile 3	−3.08 (3.60)	0.39	−15.7 (8.25)	0.06	1.04 (4.04)	0.80
	Quartile 4	3.43 (3.71)	0.36	−12.9 (7.92)	0.10	8.49 (4.16)	0.04

**Table 5:** Multivariate analysis for freedom from biochemical failure (FFBF) and freedom from distant metastasis (FFDM).

	FFBF		FFDM	
	HR (95% CI)	*p*-Value	HR (95% CI)	*p*-Value
Model 1—all men				
Treatment time, median	1.48 (0.84–2.60)	0.17	1.66 (0.78–3.54)	0.18
Risk category		0.13		<0.0001
Fav int vs. low	1.85 (0.19–17.9)		-	
Unfav int vs. low	2.41 (0.31–18.9)		-	
High vs. low	3.93 (0.54–28.9)		-	
Model 2—white men				
Treatment time, median	2.81 (0.89–8.8)	0.0583	2.82 (0.77–10.4)	0.09
Risk category		0.21		0.10
Fav int vs. low	-		-	
Unfav int vs. low	-		-	
High vs. low	-		-	
Model 3—black men				
Treatment time, median	0.97 (0.48–1.95)	0.94	0.90 (0.33–2.50)	0.84
Risk category		0.33		0.0017
Fav int vs. low	2.22 (0.20–25.1)		-	
Unfav int vs. low	2.46 (0.31–19.7)		-	
High vs. low	3.77 (0.50–28.5)		-	

- not available; model output limited by low number of events, shown for comparison.

## Data Availability

To ensure compliance with the existing IRB approval letter and HIPAA compliance for patients, the full dataset cannot be made available without prior written approval from the University of Chicago Hospital IRB. Requests to access the datasets should be directed to the corresponding author.
